# Development of *In Vitro*–*In Vivo* Correlation for Upadacitinib Extended-Release Tablet Formulation

**DOI:** 10.1208/s12248-019-0378-y

**Published:** 2019-10-25

**Authors:** Mohamed-Eslam F. Mohamed, Sheryl Trueman, Ahmed A. Othman, Jian-Hwa Han, Tzuchi R. Ju, Patrick Marroum

**Affiliations:** 1 Clinical Pharmacology and Pharmacometrics, AbbVie Inc., 1 North Waukegan Road, AP31-3, North Chicago, Illinois 60064 USA; 2 Dissolution Sciences, AbbVie Inc., North Chicago, Illinois USA

**Keywords:** upadacitinib, ABT-494, *in vitro*/*in vivo* correlations (IVIVC), extended-release formulation, pharmacokinetics

## Abstract

Upadacitinib is a selective Janus Kinase 1 inhibitor which is being developed for the treatment of several inflammatory diseases including rheumatoid arthritis. Upadacitinib was evaluated in Phase 3 studies as an oral extended-release (ER) formulation administered once daily. The purpose of this study was to develop a level A *in vitro*–*in vivo* correlation (IVIVC) for upadacitinib ER formulation. The pharmacokinetics of four upadacitinib extended-release formulations with different *in vitro* release characteristics and an immediate-release capsule formulation of upadacitinib were evaluated in 20 healthy subjects in a single-dose, randomized, crossover study. *In vivo* pharmacokinetic data and *in vitro* dissolution data (USP Dissolution Apparatus 1; pH 6.8; 100 rpm) were used to establish a level A IVIVC. Three formulations were used to establish the IVIVC, and the fourth formulation was used for external validation. A non-linear IVIVC best described the relationship between upadacitinib *in vitro* dissolution and *in vivo* absorption profiles. The absolute percent prediction errors (%PE) for upadacitinib *C*_max_ and AUC were less than 10% for all three formulations used to establish the IVIVC, as well as for the %PE for the external validation formulation and the overall mean internal validation. Model was cross-validated using the leave-one-out approach; all evaluated cross-validation runs met the regulatory acceptance criteria. A level A IVIVC was successfully developed and validated for upadacitinib ER formulation, which meets the FDA and EMA regulatory validation criteria and can be used as surrogate for *in vivo* bioequivalence.

**Electronic supplementary material:**

The online version of this article (10.1208/s12248-019-0378-y) contains supplementary material, which is available to authorized users.

## Introduction

Upadacitinib (ABT-494) is a novel, selective Janus kinase (JAK) 1 inhibitor that potently inhibits JAK 1, but is less potent against other JAK isoforms [1]. In addition to several normal physiological functions, JAKs play an essential role in the signaling of numerous cytokines involved in inflammatory disorders and inhibition of JAKs can provide approach for the treatment of patients with chronic systemic inflammatory diseases [2–4]. Upadacitinib is being developed for the treatment of several inflammatory diseases, including rheumatoid arthritis (RA), as the enhanced selectivity of upadacitinib against JAK 1 may offer an improved benefit-risk profile compared to less selective JAK inhibitors [5,6]. Upadacitinib recently demonstrated efficacy in five global Phase 3 trials in subjects with rheumatoid arthritis [7–10] and is currently under regulatory review by different global regulatory agencies for treatment of moderate-to-severe rheumatoid arthritis.

Upadacitinib pharmacokinetics was characterized in healthy subjects following the administration of the immediate-release (IR) and the extended-release (ER) formulations [11,12]. The extended-release (ER) tablet formulation of upadacitinib (the to-be-marketed formulation) was developed with the objective of decreasing the peak-to-trough fluctuations in plasma concentrations with once-daily dosing. Under fasting conditions, peak plasma concentrations of upadacitinib were reached within 2 h of administration of the ER tablet [12]. Upadacitinib plasma exposures were dose-proportional over the range of IR and ER doses evaluated in clinical studies; this encompassed doses ranging from 1 to 48 mg using the IR formulation and 7.5 to 45 mg using the ER formulation [11–14]. The bioavailability of the ER formulation used in Phase 3 studies was estimated to be 76% relative to the IR formulation [12,15]. Upadacitinib 15 mg QD and 30 mg QD using the ER formulation (the doses evaluated in Phase 3 RA studies) provide equivalent daily AUC and comparable *C*_max_ and *C*_min_ to 6 mg and 12 mg BID using the IR formulation under fasting conditions [12]. Based on *in vitro* assessments, upadacitinib has been shown to be highly permeable and highly soluble at clinically relevant doses across the pH range of 1 to 7.5 (data on file at AbbVie). Based on *in vitro* assessments, upadacitinib is considered to be a class I drug according to the Biopharmaceutics Classification System [16]. The release-controlling polymer used in the upadacitinib ER formulation is hydroxypropyl methyl cellulose (HPMC), which forms a gel layer during dissolution and controls drug release through diffusion of the drug molecule and erosion of polymer chains.

Throughout the life-cycle of a drug product, the need often arises to change some aspects of the formulation, the manufacturing process, or the manufacturing site. Some post-approval changes require the need to demonstrate bioequivalence between the modified and the marketed formulation through an *in vivo* clinical study that is adequately powered to demonstrate bioequivalence [17,18]. Further, establishing *in vivo*–*in vitro* correlation (IVIVC) can allow for the prediction of the plasma concentration time profile of a formulation without having to conduct *in vivo* bioavailability studies, thus saving time and costs and avoiding the need to administer a drug to healthy volunteers [19]. The availability of a predictive IVIVC can facilitate the establishment of clinically meaningful dissolution specifications for release and reduce the number of *in vivo* bioavailability studies which may be needed to approve and maintain a drug product on the market [17,18].

In the present study, the *in vitro* dissolution and *in vivo* pharmacokinetics for four ER formulations (30 mg strength) with various *in vitro* release profiles, including the proposed commercial formulation, were evaluated relative to a 24-mg dose of the immediate-release (IR) capsule formulation. The methodology for the evaluation and establishment of a non-linear level A IVIVC for upadacitinib ER formulations are described.

## Materials and Methods

### Formulations

Four ER formulations (A, B, C, and D) were developed with identical amounts of upadacitinib (30 mg) but varying amounts of the release-controlling polymer HPMC. Three formulations (Formulation A, Formulation B, and Formulation D) contained 10%, 15%, and 35% HPMC, respectively. The fourth formulation evaluated (Formulation C) contained 20% HPMC as a prototype for the planned commercial formulation. A single 24-mg dose of upadacitinib (2 × 12 mg IR upadacitinib capsules) was used as the reference formulation in the *in vivo* clinical study.

### *In Vitro* Dissolution Study

A USP Dissolution Apparatus 1 operating at a rotating speed of 100 rpm was used to generate the *in vitro* dissolution profiles. The dissolution medium was 900 mL of 0.05 M phosphate buffer (pH 6.8). An automatic sampler collected 1.5 mL of each of the sample at multiple time points (0, 1, 2, 4, 6, 8, 10, 12, 16, 20, 24 h), and the samples were then analyzed with high-performance liquid chromatography (HPLC). Several additional dissolution methods were also evaluated (Supplemental Table 1) in an attempt to mimic the deconvolved *in vivo* profiles and to establish a linear IVIVC (methods included different pH media, stirring speeds, dual-pH conditions, dual-RPM conditions, and use of surfactants).

### *In Vivo* Bioavailability Study

A Phase 1, single-dose, open-label study conducted according to a five-period, randomized, crossover design was used to characterize the bioavailability of four upadacitinib ER tablet formulations with different dissolution release rates relative to upadacitinib immediate-release capsules under fasting conditions. The study was conducted at PPD Development (Austin, TX, USA), and subjects were confined to the study site and supervised for approximately 21 consecutive days. Adult male subjects (*N* = 20) in general good health were selected to participate in the study. All procedures performed in studies involving human participants were in accordance with the ethical standards of the institutional and/or national research committee (Salus IRB, Austin, TX) and with the 1964 Helsinki Declaration and its later amendments or comparable ethical standards. Informed consent was obtained from all individuals included in the study prior to any study-related procedure. Enrolled subjects were randomly assigned in equal numbers to one of five sequences of regimens A, B, C, D, and E consisting of five periods. In each of the five periods, a single dose of upadacitinib was taken orally with approximately 240 mL of water after a fasting for a minimum of 10 h and at least 4 h before lunch. Each dose was separated by a washout interval of 4 days. Blood samples for upadacitinib assay were collected into dipotassium ethylenediaminetetraacetic acid-containing collection tubes prior to dosing (0 h) and at 0.25, 0.5, 1, 1.5, 2, 3, 4, 6, 8, 10, 12, 16, 24, 36, 48, and 72 h after dosing in each period. Plasma concentrations of upadacitinib were determined using a validated liquid chromatography method with tandem mass spectrometry [11]. The lower limit of quantitation (LLOQ) for upadacitinib was established at 0.05 ng/mL; the assay coefficient of variation (%CV) was ≤ 8.9%, and the mean absolute bias was ≤ 4.1%.

### Upadacitinib Pharmacokinetic Parameters

Pharmacokinetic parameters were estimated using non-compartmental methods in Phoenix® Version 7.0 (Pharsight, A Certara® Company, St. Louis, MO, USA). The maximum observed plasma concentration (*C*_max_) of upadacitinib and time to *C*_max_ (*t*_max_) values were determined directly from the plasma concentration versus time data for each subject. The apparent terminal phase elimination rate constant (β, BETA) was obtained from the slope of the least squares linear regression of the logarithms of the plasma concentration versus time data from the terminal log-linear phase of the profile. The terminal phase elimination half-life (t_1/2_) was calculated as ln (2)/β. The area under the plasma concentration versus time curve from zero to the last measurable concentration (AUC_t_) and area under the plasma concentration versus time curve from zero to infinity (AUC_inf_) were calculated by the linear trapezoidal method.

### Development of *In Vitro*–*In Vivo* Correlation (IVIVC)

A two-stage procedure was employed to develop a level A IVIVC: *in vivo* plasma concentration versus time profiles were first deconvolved followed by correlation of the fraction of drug absorbed and the fraction of drug dissolved. All numerical IVIVC analyses were conducted using IVIVC Toolkit® within Phoenix®.

The following steps were followed to develop the IVIVC model:

1. The *in vitro* dissolution time profile for each of the four formulations were fitted to a Hill (Eq. 1) or Weibull functions (Eq. 2).

1 $$ {F}_{\mathrm{diss},\mathrm{vitro}}(t)=\frac{F_{\mathrm{inf}}\ast {\mathrm{t}}^b}{{\mathrm{MDT}}^b+{t}^b} $$

where *F*_diss, vitro_ is the fitted fraction dissolved at time *t*, *F*_inf_ is the fraction dissolved at time infinity—fixed to 1, MDT is the mean dissolution time (hours), and *b* is the slope factor.

2 $$ {F}_{\mathrm{diss},\mathrm{vitro}}\left(\mathrm{t}\right)={F}_{\mathrm{inf}}\ast \left(1-\mathit{\exp}\left[-{\left(\frac{t}{\mathrm{MDT}}\right)}^b\right]\right) $$

The equation to use was selected based on adequacy of fitting the *in vitro* dissolution data and the Akaike Information Criteria (AIC) from each equation [20].

1. Individual unit impulse response (UIR) parameters were generated using the IR formulation *in vivo* data. The individual UIR parameters were estimated by fitting a maximum of three polyexponential function with a time lag to each individual profile from the IR formulation and choosing the one which best fits the profile based on the AIC with a uniform weighting scheme. Since the reference formulation was an IR formulation, the “strip K_a_” approach was followed to ensure that the UIR is decoupled from the absorption process as previously described [21]. This assumes that absorption is truly first-order and fits a model that is n-compartment poly exponential. Within the model, the maximum number of UIR exponentials was set to 3. Additionally, a lag time (*T*_lag_) was incorporated into the model to allow for a time lag in absorption. The adequacy of the selected model was determined through evaluating the agreement between model-predicted and observed upadacitinib plasma concentration versus time profiles.
2. The fraction of upadacitinib dose absorbed *in vivo* (*F*_a_) from each of the four upadacitinib ER formulations relative to the IR formulation was estimated by numerical deconvolution of the observed plasma concentration versus time profiles against the UIR from the reference IR formulation of upadacitinib. Exploratory plots were generated to evaluate the relationship between *F*_a_ and *F*_diss, vitro_ as well as between the *in vitro* dissolution time (*T*_vitro_) and *in vivo* absorption time (*T*_vivo_).
3. Several linear and non-linear models were evaluated for correlation between the fraction dissolved versus time and fraction absorbed versus time profiles of upadacitinib. Three formulations (formulation A, C, and D) were used to establish the IVIVC, and the fourth formulation (formulation B) was used for external validation. Default linear models in Phoenix IVIVC toolkit which employ absorption scale, time scale, and/or time shift for different dissolution conditions were initially evaluated. However, all the evaluated linear models demonstrated under-prediction for upadacitinib *C*_max_ and a linear IVIVC could not be established. A user-specified non-linear IVIVC was evaluated by fitting a non-linear *E*_max_ model to scale the *T*_vitro_ to the *T*_vivo_, as shown in Eq. 2. After implementing non-linear time scaling, no additional scaling factor was used between *F*_a_ and *F*_diss, vitro_.

3 $$ {T}_{\mathrm{vitro}}=\left[\frac{\left(A1\ast {T}_{\mathrm{vivo}}\right)}{\left(A2+{T}_{\mathrm{vivo}}\right)}\right]-B2 $$

where *A*1, *A*2, and *B*2 are constants characterizing the *E*_max_ relationship between *T*_vitro_ and *T*_vivo._

### Validation of the IVIVC

The developed IVIVC relationship was used to predict *F*_a_ (*F*_a, pred_) corresponding to *F*_diss, vitro_ at different time points for each formulation. The *F*_a, pred_ for each of the four ER formulations and the individual UIRs from the IR capsule formulation were used as the input function for convolution to generate individual predicted plasma concentration versus time profiles for the different ER formulations. The predicted pharmacokinetic parameters, *C*_max_, AUC_last_, and AUC_inf_ were estimated using non-compartmental analysis within the Phoenix IVIVC toolkit.

The prediction error, %PE, was calculated for AUC_inf_ and *C*_max_ for each ER formulation as:

$$ \left[\left(\mathrm{predicted}\hbox{--} \mathrm{observed}\right)/\mathrm{observed}\right]\times 100\% $$

Additionally, a cross-validation was conducted using the leave-one-out approach. The final IVIVC model was re-run using each of the four extended-release formulations (A, B, C, and D) as an external validation formulation and the remaining three formulations for model building and internal validation. The %PE was calculated for internal and external validation formulations for each of the cross-validation runs.

The predictive ability of the IVIVC was assessed for the numerical IVIVC through evaluating each method’s internal and external predictability per FDA and EMA guidance documents [17,18]. To establish the internal predictability of the IVIVC, an average absolute % PE of 10% or less for *C*_max_ and AUC_inf_ was required. In addition, the absolute % PE for each individual formulation was required not to exceed 15%. For external validation, the absolute %PE was required not to exceed 10% for *C*_max_ and AUC_inf_.

## Results

### *In Vitro* Dissolution Study

**Fig. 1:**
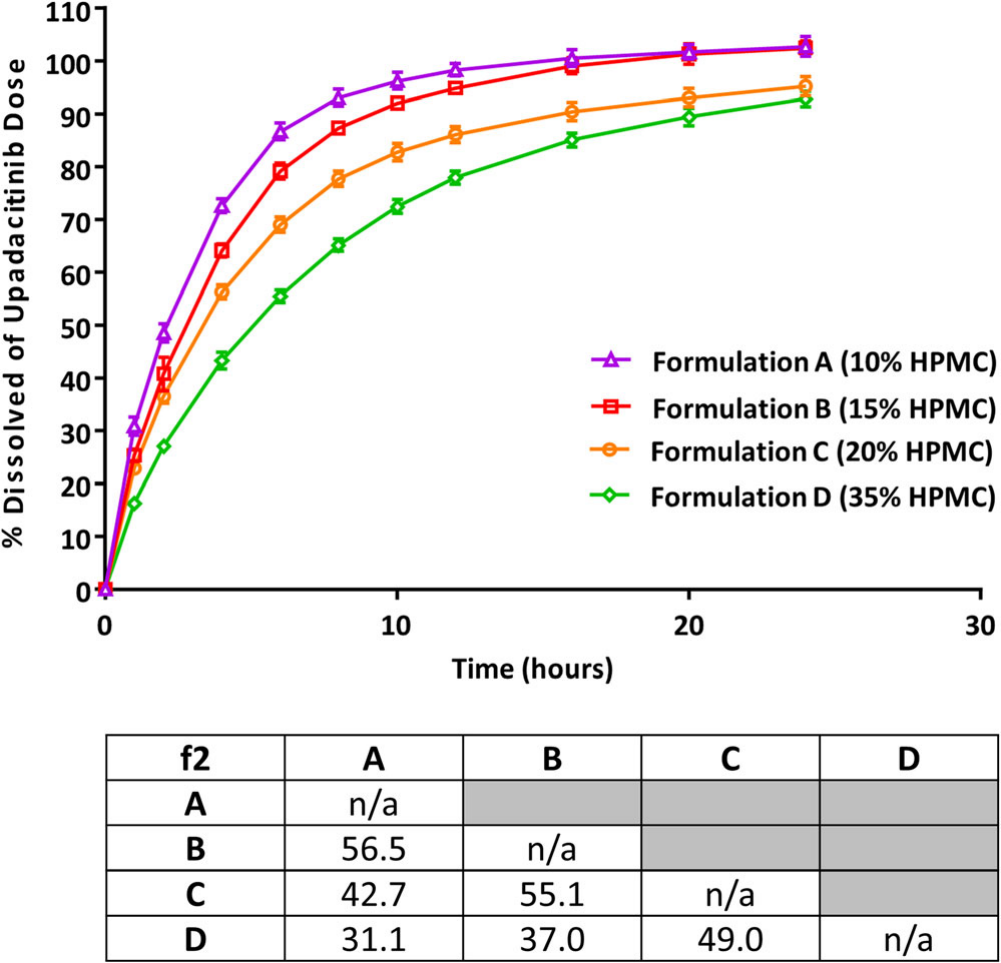
Cumulative percent dissolved (mean ± SD) versus time profiles for upadacitinib ER formulations containing 10% HPMC (formulation A), 15% HPMC (formulation B), 20% HPMC (formulation C; target formulation), and 35% HPMC (formulation D). F2 values comparing the four formulations are presented

The *in vitro* drug-release profiles for the four upadacitinib ER formulations using the selected method (USP App 1 at pH 6.8) are shown in Fig. 1 along with f2 values comparing the different formulations to each other. Formulations A, B, C, and D released at least 80% of drug by 6, 8, 10, and 16 h, respectively. To evaluate the possibility of linear IVIVC, additional dissolution conditions were evaluated, but a linear IVIVC that could meet the FDA required predictions error criteria was not established (Supplemental Table 1). A Weibull function (Eq. 2) was used to fit the *in vitro* dissolution data as it provided better fit compared to a Hill function (Eq. 1) and resulted in lower AIC values by 10 to 35 points (for formulations A, B, C, and D, AIC values were − 61, − 63, − 62, and − 84, respectively, for Weibull function and − 40, − 53, − 39, and − 48, respectively, for the Hill function).

### *In Vivo* Bioavailability Study Results

**Fig. 2:**
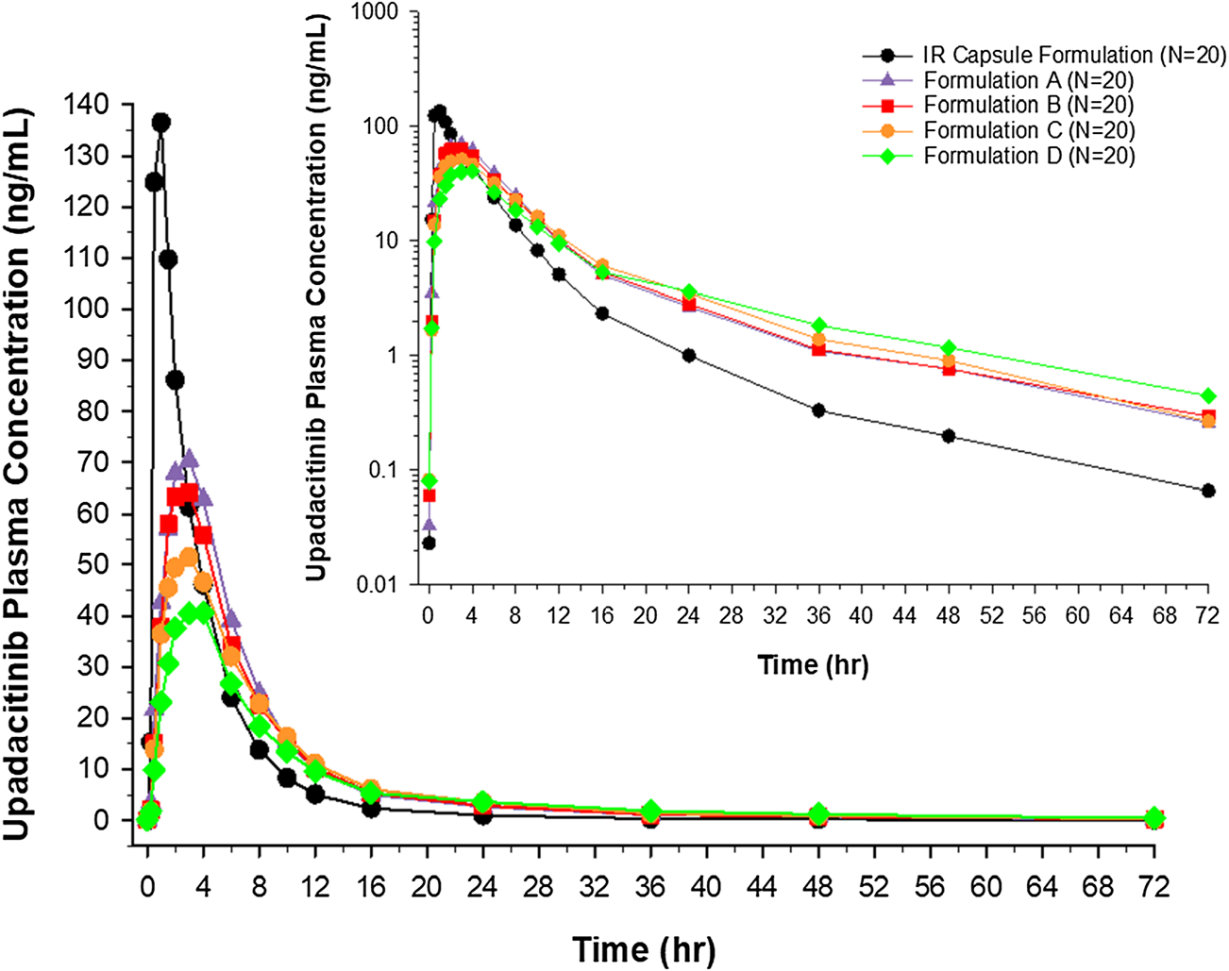
Mean upadacitinib plasma concentration versus time profiles following administration of IR capsule and ER tablet formulations of upadacitinib. ER formulations of upadacitinib contained 10% HPMC (formulation A), 15% HPMC (formulation B), 20% HPMC (formulation C; target formulation), and 35% HPMC (formulation D). *Insert*: Log-linear scale of mean upadacitinib plasma concentration versus time profiles following administration of IR capsule and ER tablet formulations of upadacitinib

**Fig. 3:**
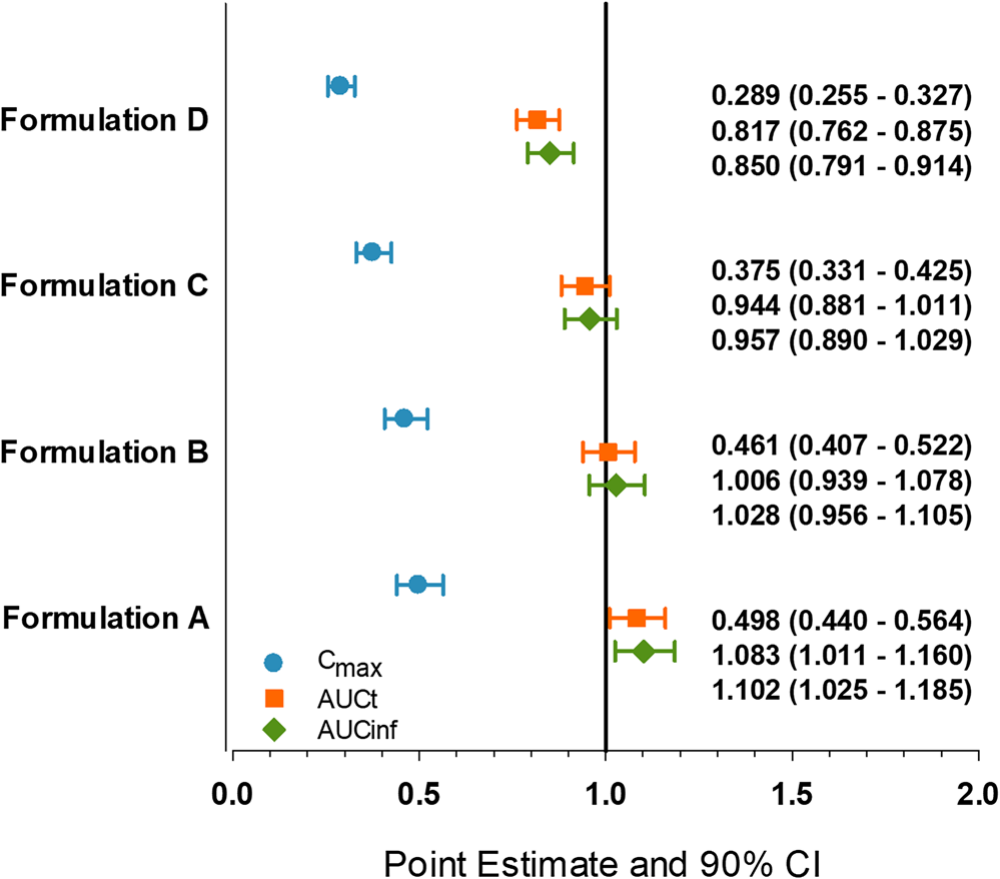
Point Estimates and 90% confidence intervals for the bioavailability of upadacitinib following administration of single doses of upadacitinib 30 mg extended-release tablets with different release rates relative to single 24 mg dose of the upadacitinib immediate-release capsules

The mean plasma concentration versus time profiles from the *in vivo* study for the ER formulations A, B, C, and D and the IR formulations are presented in Fig. 2. The evaluated ER formulations (A, B, C, and D) showed central ratios of upadacitinib AUC_inf_ and *C*_max_ relative to the reference IR capsule formulation in the rank order of their *in vitro* release rate and HPMC content (Fig. 3). The ratios of central values for upadacitinib *C*_max_ were 0.5, 0.46, 0.38, and 0.29 for formulations A, B, C, and D, respectively, relative to the IR capsule formulation. The ratios of the central values for upadacitinib AUC_inf_ were 1.1, 1.0, 0.96, and 0.85 for formulations A, B, C, and D, respectively, relative to the IR capsule formulation. The pharmacokinetic parameters of upadacitinib following administration of single doses of upadacitinib IR capsules (24 mg) and ER tablets (30 mg) are described in Table I. Median *T*_max_ of upadacitinib was 2.0, 2.5, 3.0, and 3.0 h for ER formulations A, B, C, and D, respectively, as compared to 1.0 h for the IR formulation. Upadacitinib terminal half-life was similar across the ER formulations and comparable to that of the IR formulation (approximately 10 to 13 h).

**Table I:** Pharmacokinetic Parameters of Upadacitinib Following Administration of Single Doses of Upadacitinib IR Capsules (24 mg) and ER Tablets (30 mg) with Different Release Rates Under Fasting Conditions

			Formulation		
Pharmacokinetic Parameters (units)	IR Capsules UPA 24 mg (*N* = 20)	ER formulation A 10% HPMC UPA 30 mg (*N* = 20)	ER formulation B 15% HPMC UPA 30 mg (*N* = 20)	ER formulation C* 20% HPMC UPA 30 mg (*N* = 20)	ER formulation D 35% HPMC UPA 30 mg (*N* = 20)
*C*_max_(ng/mL)	159 ± 45.7	79.3 ± 24.3	72.6 ± 19.7	59.5 ± 16.7	46.2 ± 14.7
*T*_max_^a^ (h)	1.0 (0.5–2.0)	2.0 (0.5–4.0)	2.5 (1.5–4.0)	3.0 (1.0–6.0)	3.0 (1.0–6.0)
AUC_t_ (ng · h/mL)	507 ± 85.0	549 ± 89.5	515 ± 114	487 ± 120	422 ± 106
AUC_inf_ (ng · h/mL)	510 ± 85.1	562 ± 89.3	529 ± 111	497 ± 121	443 ± 113
t_1/2_^b^ (h)	10.2 (6.92)	12.2 (8.24)	10.7 (7.65)	9.96 (6.09)	12.5 (8.06)

*UPA* upadacitinib

*Target formulation

^a^Median (minimum through maximum)

^b^Harmonic mean (pseudo-standard deviation)

### IVIVC Model

**Fig. 4:**
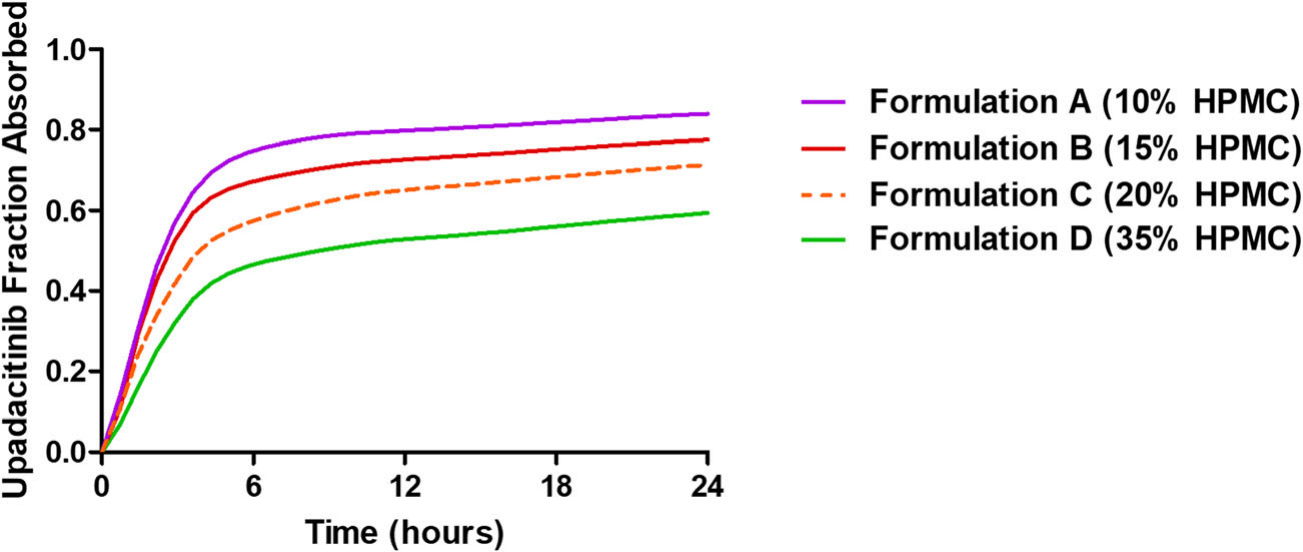
Mean *in vivo* absorption versus time profile of upadacitinib extended-release formulations based on numerical deconvolution

**Fig. 5:**
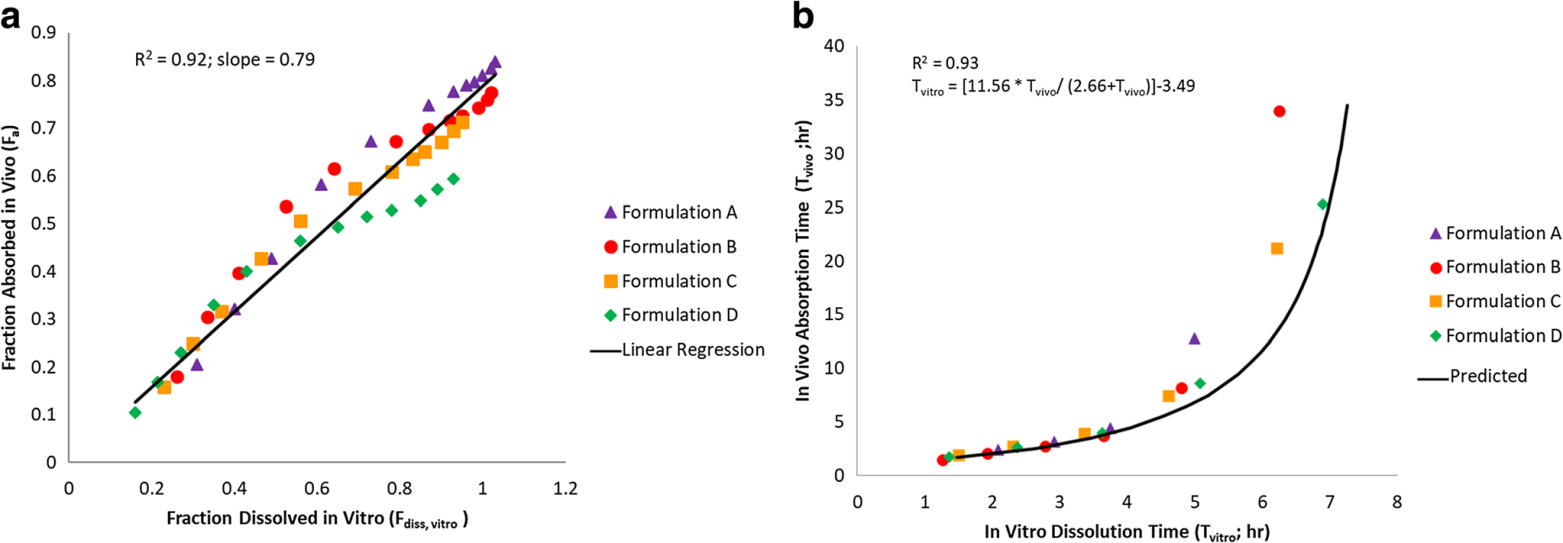
Correlation between **a** the observed fraction absorbed and the fraction dissolved and **b** *in vivo* absorption time and *in vitro* dissolution time for upadacitinib ER formulations

The mean fraction of upadacitinib dose absorbed versus time profiles are presented in Fig. 4 for the different extended-release formulations based on deconvolution. Exploratory plots for the fraction of upadacitinib *F*_a_ and *F*_diss, vitro_ as well as between *T*_vivo_ and *T*_vitro_ are presented in Fig. 5. Based on the exploratory plots, the relation between *F*_a, obs_ and *F*_d, obs_ is linear (*R*^2^ = 0.92; Fig. 5a); however, there is a clear non-linearity between *T*_vivo_ and *T*_vitro_ (Fig. 5b). Therefore, default linear models with Phoenix IVIVC toolkit could not be used to establish an IVIVC for upadacitinib ER formulations. The relationship between *T*_vitro_ and time *T*_vivo_ was best described by a non-linear *E*_max_ function with intercept (Eq. 2) as described in methods and presented in Fig. 5b. The mean estimates (and standard error of the estimates) of the IVIVC parameters (Eq. 2) were 11.6 (0.42) h for A1, 2.66 (0.28) h for A2, and 3.49 (0.47) h for B2.

### Internal and External Validation

Summary of the observed and predicted upadacitinib AUC_inf_ and C_max_ for each of the three formulations used to develop the IVIVC (formulations A, C, and D) as well as for the external validation formulation (formulation B) are presented in Table II. The average %PE was less than 10% for both AUC_inf_ and *C*_max_ for the ER formulations (A, C, and D). The %PE for each of the individual ER formulations A, C, and D were also well below 15% for both AUC_inf_ and *C*_*max*_. In addition, the external %PE for ER formulation B was less than 10% (Table II). These results demonstrate that the established IVIVC meets the predefined internal and external validation criteria.

**Table II:** Results of the Internal and External Validation for Upadacitinib IVIVC

Internal validation formulations													External validation formulation		
	Formulation A 10% HPMC			Formulation C (target formulation) 20% HPMC			Formulation D 35% HPMC			Average for internal validation			Formulation B15% HPMC		
Parameter	Obs.	Pred.	%PE	Obs.	Pred.	%PE	Obs.	Pred.	%PE	Obs.	Pred.	Absolute %PE	Obs.	Pred.	%PE
*C*_max_ (ng/mL)	75.9	78.6	3.55	57.2	58.0	1.40	44.0	44.6	1.22	57.6	58.8	2.06	70.2	68.2	− 2.85
AUC_last_ (h · ng/mL)	532.0	561.8	5.60	463.2	462.6	− 0.13	400.5	389.1	− 2.84	462.1	465.9	2.86	494.1	524.1	6.08
AUC_inf_ (h · ng/mL)	545.1	565.3	3.70	472.9	466.9	− 1.27	420.0	393.8	− 6.24	476.6	470.2	3.74	508.2	528.3	4.00

Note: Values are reported as the geometric mean

Results from the cross-validation using the leave-one-out approach are presented in Table III. All cross-validation runs met the acceptance criteria (%PE for each formulation < 15%; average internal validation %PE < 10%; external validation %PE < 10%).

**Table III:** Cross-Validation Results for the Non-Linear IVIVC Model Using the Leave-One-Out Approach

Validation Initial correlation estimates Final parameter estimates	Formulation	Parameter^a^	Predicted	Observed	%PE
Model developed with formulations A, C, and D. Formulation B used for external validation Initial estimates: A1: 15 h; A2: 5 h; B2: 8 h Final Estimates: A1: 11.6 h; A2: 2.7 h; B2: 3.5 h	A	AUC_inf_	565	545	3.7
	A	*C*_max_	79	76	3.6
	C	AUC_inf_	467	473	− 1.3
	C	*C*_max_	58	57	1.4
	D	AUC_inf_	394	420	− 6.2
	D	*C*_max_	45	44	1.2
	Avg internal	AUC_inf_	470	477	3.7
	Avg internal	*C*_max_	59	58	2.1
	B	AUC_inf_	528	508	4.0
	B	*C*_max_	68	70	− 2.9
Model developed with formulations B, C, and D. Formulation A used for external validation Initial estimates: A1: 15 h; A2: 5 h; B2: 8 h Final estimates: A1: 11.6 h; A2: 2.4 h; B2: 3.7 h	B	AUC_inf_	527	508	3.7
	B	*C*_max_	69	70	− 1.9
	C	AUC_inf_	465	473	− 1.6
	C	*C*_max_	59	57	2.4
	D	AUC_inf_	392	420	− 6.7
	D	*C*_max_	45	44	2.5
	Avg internal	AUC_inf_	458	466	4.0
	Avg internal	*C*_max_	57	56	2.2
	A	AUC_inf_	565	545	3.6
	A	*C*_max_	79	76	4.4
Model developed with formulations A, B, and C. Formulation D used for external validation Initial estimates: A1: 16 h; A2: 6 h; B2: 10 h Final estimates: A1: 17.1 h; A2: 3.2 h; B2: 7.3 h	A	AUC_inf_	592	545	8.6
	A	*C*_max_	73	76	− 3.6
	B	AUC_inf_	561	508	10.4
	B	*C*_max_	63	70	− 10.2
	C	AUC_inf_	503	473	6.4
	C	*C*_max_	55	57	− 4.4
	Avg internal	AUC_inf_	551	508	8.4
	Avg internal	*C*_max_	63	67	6.0
	D	AUC_inf_	433	420	3.1
	D	*C*_max_	41	44	− 6.3
Model developed with formulations A, B, and D. Formulation C used for external validation Initial estimates: A1: 16 h; A2: 6 h; B2: 10 h Final estimates: A1: 11.6 h; A2: 2.0 h; B2: 4.2 h	A	AUC_inf_	558	545	2.4
	A	*C*_max_	79	76	4.4
	B	AUC_inf_	518	508	1.8
	B	*C*_max_	69	70	− 1.6
	D	AUC_inf_	380	420	− 9.6
	D	*C*_max_	45	44	2.9
	Avg internal	AUC_inf_	479	488	4.6
	Avg internal	*C*_max_	63	62	3.0
	C	AUC_inf_	455	473	− 3.8
	C	*C*_max_	59	57	2.6

^a^Units are ng · h/mL for AUC_inf_ and ng/mL for *C*_max_

## Discussion

A predictive level A non-linear IVIVC was established for upadacitinib ER formulation using *in vivo* and *in vitro* data for formulations containing a range of the release-controlling polymer (HPMC) of 10 to 35%. This range encompasses the target to be marketed formulation (formulation C) which contains 20% HPMC. The established IVIVC meets both the internal and external predictability per the FDA and EMA criteria and can potentially be used as surrogate for *in vivo* study if there is a need for formulation change (within the design space of the IVIVC) or manufacturing process change. Additionally, the established IVIVC enables setting the release specifications based on clinical relevance using the predicted range of upadacitinib exposures for formulations that fall within the proposed specifications. These analyses highlight the importance of evaluating non-linear models when linear IVIVC relationships cannot be established.

Non-linear IVIVC models, although less common than linear models, are considered acceptable as long as internal and/or external validation demonstrates adequate predictability of the IVIVC and that the same non-linear time scale is used for all formulations [17–19]. Evaluation of linear models for upadacitinib ER formulation consistently resulted in under-prediction of upadacitinib *C*_max_, (data not shown) suggesting slower dissolution *in vitro* than *in vivo* dissolution that was not adequately accounted for using a linear time scaling factor. This was also the case for various other *in vitro* test conditions evaluated to mimic the *in vivo* conditions (Supplemental Table 1). In general, potential causes for non-linearity in IVIVC can be expected to be the lack of uniformity in absorption of a drug throughout the gastrointestinal tract, possible saturation of transporters or first-pass metabolic pathways, or the nature of the formulation [22,23].

Upadacitinib is a highly soluble and highly permeable drug that exhibits linear pharmacokinetic characteristics over the range of doses that have been studied (up to a dose of 45 mg using the extended-release formulation and up to 48 mg using the immediate-release formulation). Given the high permeability and high solubility of upadacitinib, intestinal transporters are not expected to have clinically relevant role in upadacitinib disposition *in vivo* and no data suggest lack of uniformity in absorption throughout the gastrointestinal tract in humans. Therefore, it is likely that the nature of the formulation and how it releases the drug *in vivo* is driving the non-linear relationship between *T*_vitro_ and *T*_vivo_. Analysis of deconvolved *in vivo* absorption profiles (Fig. 4) demonstrated a common two-stage profile for all four tested formulations: rapid early absorption over the first 4 to 5 h, followed by slower absorption afterward. Drug release from gel-forming hydrophilic matrices (such as that used in this upadacitinib controlled release formulation) has been known to come from two mechanisms: diffusion of drug molecules across the gel layer formed on the surface of the tablet and erosion of the polymer on the tablet surface (or the outer layer of the gel). The dissolution conditions tested *in vitro* (including those that used two different pHs or RPMs in the same test; Supplemental Table 1) were unable to mimic the time course of the release *in vivo* for all formulations; thus, establishing a linear IVIVC relationship was not feasible. Only a non-linear IVIVC with prediction errors meeting the acceptance criteria could be achieved no matter what dissolution condition was used. An important consideration for the development and validation of this non-linear correlation is that the model fitted to correct for the time scale difference between the *in vitro* data and the *in vivo* data is the same for all the formulations tested and is independent of the formulation or its release rate.

It is worth noting that the relationship between *T*_vitro_ and *T*_vivo_ appeared linear up to approximately 8 h *in vivo*. An approach that is alternative to the use of non-linear model would be to establish a linear IVIVC with *in vivo* cutoff time (*t*_cutoff_). However, use of *t*_cutoff_ implies ignoring collected *in vivo* and *in vitro* data after the selected *t*_cutoff_. Additionally, Phoenix software that does not allow estimating the optimal *t*_cutoff_ value and *t*_cutoff_ will need to be selected as an arbitrary value based on observed data. Both of the aforementioned points can potentially introduce bias in the analysis when *t*_cutoff_ is used. We opted to use a non-linear IVIVC approach rather than use of linear IVIVC with *t*_cutoff_ to be able to use the totality of the data thus to avoid ignoring any *in vitro* or *in vivo* data and to allow the software to estimate the best-fitting parameters for the non-linear relationship based on the data.

The IVIVC model was developed while a Phase 3 study which evaluated the safety and efficacy of upadacitinib doses of 15 mg and 30 mg QD using the ER formulation was ongoing. The upadacitinib ER formulation is considered proportionally similar between the 15 and 30 mg strengths, and upadacitinib plasma exposures are linear over a wide range of IR and ER doses [8,15]. Therefore, the IVIVC, developed using the highest clinically relevant strength at the time of conducting the analysis (30 mg strength), is considered applicable to the 15 mg strength.

The robustness of the model was evaluated through cross-validation using the leave-one-out approach; all cross-validation runs meet the acceptance criteria (%PE for each formulation < 15%; average internal validation %PE < 10%; external validation %PE < 10%). This assessment, although not a requirement per regulatory guidance documents, demonstrate robustness of the IVIVC and that it is not sensitive to data from a specific formulation. With a validated IVIVC, an *in vitro* test can potentially serve as a surrogate for bioavailability testing as well as a tool to screen formulations and set the dissolution and drug-release acceptance criteria.

## Conclusion

A robust non-linear level A correlation that meets the FDA and EMA validation criteria for both internal and external predictability was established for upadacitinib ER formulation. This IVIVC can be used as surrogate for bioequivalence studies in case of future formulation changes that are covered by the IVIVC release rates tested. This correlation will enable the setting of clinically meaningful dissolution specifications based on acceptable differences in plasma concentrations corresponding to the upper and lower limit of the dissolution specifications.

## Electronic supplementary material

ESM 1 (DOCX 14 kb)

## References

[1] Parmentier JM, Voss J, Graff C, Schwartz A, Argiriadi M, Friedman M, Camp HS, Padley RJ, George JS, Hyland D, Rosebraugh M, Wishart N, Olson L, Long AJ (2018). In vitro and in vivo characterization of the JAK1 selectivity of upadacitinib (ABT-494). BMC Rheumatol.

[2] Burmester GR, Kremer JM, Van den Bosch F, Kivitz A, Bessette L, Li Y, et al. Safety and efficacy of upadacitinib in patients with rheumatoid arthritis and inadequate response to conventional synthetic disease-modifying anti-rheumatic drugs (SELECT-NEXT): a randomised, double-blind, placebo-controlled phase 3 trial. Lancet. 2018;391(10139):2503–12. 10.1016/S0140-6736(18)31115-2. 10.1016/S0140-6736(18)31115-2 29908669

[3] Leonard WJ (2001). Role of Jak kinases and STATs in cytokine signal transduction. Int J Hematol.

[4] O'Shea JJ, Plenge R (2012). JAK and STAT signaling molecules in immunoregulation and immune-mediated disease. Immunity.

[5] Kremer JM, Emery P, Camp HS, Friedman A, Wang L, Othman AA, Khan N, Pangan AL, Jungerwirth S, Keystone EC (2016). A phase IIb study of ABT-494, a selective JAK-1 inhibitor, in patients with rheumatoid arthritis and an inadequate response to anti-tumor necrosis factor therapy. Arthritis Rheum.

[6] Genovese MC, Smolen JS, Weinblatt ME, Burmester GR, Meerwein S, Camp HS, Wang L, Othman AA, Khan N, Pangan AL, Jungerwirth S (2016). Efficacy and safety of ABT-494, a selective JAK-1 inhibitor, in a phase IIb study in patients with rheumatoid arthritis and an inadequate response to methotrexate. Arthritis Rheumatol.

[7] Genovese MC, Fleischmann R, Combe B, Hall S, Rubbert-Roth A, Zhang Y, Zhou Y, Mohamed MEF, Meerwein S, Pangan AL (2018). Safety and efficacy of upadacitinib in patients with active rheumatoid arthritis refractory to biologic disease-modifying anti-rheumatic drugs (SELECT-BEYOND): a double-blind, randomised controlled phase 3 trial. Lancet.

[8] Mohamed MF, Zeng J, Marroum PJ, Song IH, Othman AA (2019). Pharmacokinetics of upadacitinib with the clinical regimens of the extended-release formulation utilized in rheumatoid arthritis phase 3 trials. Clin Pharmacol Drug Dev.

[9] Fleischmann R, Pangan AL, Song IH, Mysler E, Bessette L, Peterfy C, et al. Upadacitinib versus Placebo or Adalimumab in Patients with Rheumatoid Arthritis and an Inadequate Response to Methotrexate: Results of a Phase 3, Double-Blind, Randomized Controlled Trial. Arthritis Rheumatol. 2019. 10.1002/art.41032. 10.1002/art.41032 31287230

[10] Van Vollenhoven R, Takeuchi T, Pangan AL, Friedman A, Mohamed MF, Chen S, et al. A phase 3, randomized, controlled trial comparing upadacitinib monotherapy to MTX monotherapy in MTX-naïve patients with active rheumatoid arthritis [abstract]. Arthritis and Rheumatol. 2018;70(suppl 10).

[11] Mohamed MF, Camp HS, Jiang P, Padley RJ, Asatryan A, Othman AA (2016). Pharmacokinetics, safety and tolerability of ABT-494, a novel selective JAK 1 inhibitor, in healthy volunteers and subjects with rheumatoid arthritis. Clin Pharmacokinet.

[12] Mohamed MF, Zeng J, Marroum PJ, Song IH, & Othman AA. Pharmacokinetics of upadacitinib with the clinical regimens of the extended-release formulation utilized in rheumatoid arthritis phase 3 trials. Clin Pharmacol Drug Dev. 2019;8(2):208–216. 10.1002/cpdd.462. 10.1002/cpdd.462 PMC6585649 29688617

[13] Klunder B, Mohamed MF, Othman AA (2018). Population pharmacokinetics of upadacitinib in healthy subjects and subjects with rheumatoid arthritis: analyses of phase I and II clinical trials. Clin Pharmacokinet.

[14] Nader A, Stodtmann S, Friedel A, Mohamed MF, & Othman AA. Pharmacokinetics of upadacitinib in healthy subjects and subjects with rheumatoid arthritis, Crohn's disease, ulcerative colitis or atopic dermatitis: population analyses of Phase 1 and 2 clinical trials. 2019;In Press. 10.1002/jcph.1550 31701537

[15] Klünder B, Mittapalli RK, Mohamed M-EF, Friedel A, Noertersheuser P, Othman AA (2019). Population pharmacokinetics of upadacitinib using the immediate-release and extended-release formulations in healthy subjects and subjects with rheumatoid arthritis: analyses of phase I–III clinical trials. Clin Pharmacokinet.

[16] FDA. Guidance for Industry. Waiver of in vivo bioavailability and bioequivalence studies for immediate-release solid oral dosage forms based on a biopharmaceutics classification system. https://www.fda.gov/downloads/Drugs/GuidanceComplianceRegulatoryInformation/Guidances/UCM070246.pdf Accessed January 24 2019. 2017.

[17] FDA. Guidance for Industry. Extended release oral dosage forms: development, evaluation, and application of in vitro/in vivo correlations. US Department of Health and Human Services, Food and Drug Administration, Center for Drug Evaluation and Research (CDER). Available from: https://www.fda.gov/downloads/drugs/guidances/ucm070239.pdf Accessed January 10 2019. 1997.

[18] EMA. European Medicines Agency. Guidance on the Investigation of Bioequivalence. 2010. Available from: https://www.ema.europa.eu/en/documents/scientific-guideline/guideline-investigation-bioequivalence-rev1_en.pdf. Accessed September 16 2019.

[19] Suarez-Sharp S, Li M, Duan J, Shah H, Seo P (2016). Regulatory experience with in vivo in vitro correlations (IVIVC) in new drug applications. AAPS J.

[20] Sakamoto Y, Ishiguro M, & Kitagawa G (1986) Akaike information criterion statistics. KTK Scientific Publishers ; D. Reidel ; Sold and distributed in the U.S.A. and Canada by Kluwer Academic Publishers: Tokyo; Dordrecht; Boston; Hingham, MA.

[21] Kakhi M, Marroum P, Chittenden J (2013). Analysis of level A in vitro-in vivo correlations for an extended-release formulation with limited bioavailability. Biopharm Drug Dispos.

[22] Sirisuth N, Augsburger LL, Eddington ND (2002). Development and validation of a non-linear IVIVC model for a diltiazem extended release formulation. Biopharm Drug Dispos.

[23] Cardot JM, Davit BM (2012). In vitro-in vivo correlations: tricks and traps. AAPS J.

