# Supplementary material for: Development of In Vitro–In Vivo Correlation for Upadacitinib Extended-Release Tablet Formulation
Source: AAPS J. 2019 Oct 25;21(6):108. doi: 10.1208/s12248-019-0378-y (PMC6814631; doi:10.1208/s12248-019-0378-y)
Supplement: Supplementary file 1 — (DOCX 14 kb) [file 12248_2019_378_MOESM1_ESM.docx]

**Supplemental Table 1.** Summary of the different dissolution methods evaluated in attempt to establish a linear IVIVC for upadacitinib ER formulation.

| **Dissolution Condition Description** | **pH** | **Stirring Speed (RPM)** | **Apparatus** | **Internal Validation** | **Parameter** | **Average Absolute Internal**  **%PE** |
| --- | --- | --- | --- | --- | --- | --- |
| Single condition | pH 6.8 | 100 | App 1 | Failed | AUC_Last_  C_max_ | 5.7  31.3 |
| Single condition | pH 1.1 (0.1N HCL) | 100 | App 1 | Failed | AUC_Last_  C_max_ | 8.2  31.5 |
| Single condition | pH 4.5 | 100 | App 1 | Failed | AUC_Last_  C_max_ | 7.3  31.4 |
| Dual – pH condition | pH 1.1 (0.1N HCl) to pH 6.8 after 2 hours | 100 | App 1 | Failed | AUC_Last_  C_max_ | 5.7  31.5 |
| Dual – pH and RPM condition | pH 6.8 to pH 7.4 after 4.5 hours | 100 to 10 after 4.5 hours | App 1 | Failed | AUC_Last_  C_max_ | 5.5  30.5 |
| Dual – RPM condition with surfactant | pH 6.8 + 0.3% SDS to 0.17% SDS after 4.5 hours | 100 to 25 after 4.5 hours | App 1 | Failed | AUC_Last_  C_max_ | 5.7  30.7 |
| Dual – pH and RPM condition | pH 1.1 to pH 6.8 after 4.5 hours | 100 to 25 RPM | App 1 | Failed | AUC_Last_  C_max_ | 6.7  32.1 |
| Dual – RPM condition | pH 4.0 | 100 to 10 RPM | App 1 | Failed | AUC_Last_  C_max_ | 7.1  32.4 |
| Dual – pH and RPM condition | pH 4.0 to pH 6.8 (Hi Salt) after 4.5 hours | 100 to 10 RPM | App 1 | Failed | AUC_Last_  C_max_ | 4.7  29.8 |
| Dual Condition – pH and RPM condition | pH 2 to pH 7.5 (High Salt) after 4.5 hours | 100 to 10 after 4.5 hours | App 1 | Failed | AUC_Last_  C_max_ | 4.0  28.1 |
